# Exploring effects of resilience-focused debriefing on reflection and teamwork in interprofessional simulation-based education – a mixed method study

**DOI:** 10.1186/s41077-025-00398-4

**Published:** 2025-12-19

**Authors:** Torben Nordahl Amorøe, Hans Rystedt, Lena Oxelmark, Peter Dieckmann, Karin Jonsson, Cecilia Escher, Johan Creutzfeldt, Paulin Andréll

**Affiliations:** 1 Department of Anesthesiology and Intensive Care Medicine, Institute of Clinical Sciences, Sahlgrenska Academy, University of Gothenburg, Gothenburg, Sweden; 2 Simulation Centre, Department of Research, Development, Education and Innovation, Sahlgrenska University Hospital, Region Västra Götaland, Diagnosvägen 10, Gothenburg, 416 85 Sweden; 3 Institute of Health and Care Sciences, Sahlgrenska Academy, University of Gothenburg, Gothenburg, Sweden; 4 Copenhagen Academy for Medical Education and Simulation (CAMES), Center for Human Resources, Capital Region of Denmark, Herlev Hospital, Herlev, Denmark; 5 Department of Quality and Health Technology, University of Stavanger, Stavanger, Norway; 6 Department of Public Health, University of Copenhagen, Copenhagen, Denmark; 7 Department of Nursing, Umeå University, Umeå, Sweden; 8 Department of Clinical Science, Intervention, and Technology, Karolinska Institutet, Stockholm, Sweden; 9 Center for Advanced Medical Simulation and Training, Karolinska University Hospital, Stockholm, Sweden; 10 Department of Anaesthesiology, Intensive Care Medicine and Pain Medicine, Sahlgrenska University Hospital, Gothenburg, Region Västra Götaland Sweden

**Keywords:** Complexity, Debriefing, Interprofessional education, Learning from success, Patient safety, Resilient healthcare, Safety II, Patient simulation, Team performance

## Abstract

**Background:**

Interprofessional simulation-based education (IPSE) holds the potential to prepare healthcare students to handle the complexity of healthcare. However, complexity and resilience are traditionally not addressed deliberately in IPSE. The aim of this study was to explore the effect of resilience-focused debriefing (RFD) that addresses complexity and resilience, on reflection and teamwork in IPSE for pre-graduate healthcare students.

**Methods:**

In a convergent mixed methods intervention study, 149 nursing and medical students in their last semester participated in a full-day IPSE course with five progressively challenging scenarios. Fifteen facilitators were instructed to use RFD. Qualitative date, comprised of transcripts from nine debriefings, were analysed using topic analysis. An intervention check was performed to assess the use of RFD. Quantitative data comprised pre-post ratings of team performance in videorecorded scenarios (1 and 5) from 18 groups using the Team Emergency Assessment Measure (TEAM). Additionally, a study-specific rating scale was employed to assess the extent of participants’ perceived challenges during scenarios.

**Results:**

RFD helped facilitators to guide the students’ attention to the complexity of teamwork and how to manage such complex situations successfully by adapting crisis resource management principles and performing resilient actions (e.g., attunement, adaptive leadership), both as individuals and as teams. Applying RFD brought the students’ attention to how they were able to succeed despite the difficulties they encountered. Although the assessed team performance was on an acceptable level, students initially had difficulties in recognizing and learning from actions that led to successful outcomes. The significant decrease in the degree of challenges experienced suggests that students developed a greater tolerance for complexity. Nevertheless, the quantitative data showed that there was no pre-post difference in team performance as assessed by TEAM.

**Conclusions:**

RFD can be used to increase healthcare students’ attention to the complexity of interprofessional teamwork in acute dynamic situations and help them recognize and learn from both successful actions and overcoming challenging situations. Although we did not find a significant gain in team performance, the integrated results suggest that RFD may potentially improve interprofessional teamwork. Further research is warranted to develop instruments measuring team performance that are sensitive to various aspects of resilience, as well as to deepen the understanding of RFD in the simulation-based education.

**Supplementary Information:**

The online version contains supplementary material available at 10.1186/s41077-025-00398-4.

## Background

In healthcare education, the main goal of interprofessional simulation-based education (IPSE) is to prepare students for clinical work [1, 2]. However, simulation-based education rarely deliberately addresses how to manage the complexity of everyday clinical work in healthcare settings [3, 4].

In everyday clinical work, healthcare staff navigate a complex system that is rarely stable and requires adjustments [5]. Such systems are characterized by features such as emergence (unexpected behaviours arise from interactions among parts), inter-dependability (components are mutually reliant, so changes in one affect others), self-organization (a system adapts and structures itself without centralized control), and non-linearity (relationships are not proportional or tractable—small actions can lead to large, unpredictable outcomes) [6–8]. Healthcare teams are complex systems in themselves [9], and each member of the team needs to adapt to planned and unplanned changes, dynamic events, and disturbances [8]. In this context, resilience is described as successful adjustments to emergent events [10] and has been defined as “the capacity to adapt to challenges and changes at different system levels, to maintain high quality care” [11]. Thus, resilience is described to take place at both systems, team and individual levels. This research study is primarily concerned with the team level i.e. team resilience. Resilience in this context may, for example, be awareness of team dynamics and all kinds of adjustments to the unexpected, patient’s sudden deterioration, emerged uncertainty about leadership or communication. Furthermore, resilience is thought to be connected to Safety-II, which focuses on understanding and reinforcing what goes well in order to achieve successful outcomes. Safety-I, in contrast, concentrates on analysing what goes wrong in order to eliminate errors [10].

To meet such challenges, healthcare staff need to be able to effectively collaborate in interprofessional teams [1]. IPSE seeks to teach and train interprofessional teamwork and collaboration to assure patient safety [12]. This training involves interprofessional teams managing medical and nursing challenges in full-scale simulation scenarios, followed by structured reflections termed debriefings. The purpose of the debriefings is to help the participants make sense of their experiences and to increase their capabilities on different levels, including, for example, the diagnosis and treatment of the patient, situational awareness, decision-making, task management, and teamwork [13]. Thus, debriefing is seen as a crucial component of IPSE – often expressed as the “heart and soul” of simulation-based training [14].

In IPSE, training usually focuses on how to manage dynamic events in the acute care of patients [15]. This is primarily achieved by training adherence to a number of standards, algorithms, and guidelines. However, the complexity of interprofessional teamwork in acute situations and the need for resilience are rarely addressed deliberately [3, 4, 7]. For example, attention is rarely intentionally put on adaptation, alternative solutions, and recovering from setbacks. In two previous studies, we explored debriefing practice and developed debriefing principles [16] and specific techniques [17] that aimed to address complexity and resilience in IPSE, termed resilience-focused debriefing (RFD. The basis for the development of these techniques was theories of complexity, resilient healthcare, Safety-II, and solution-focused and appreciative inquiry approaches [18, 19].

Until now the effect of debriefings that specifically address complexity and resilience in IPSE has not been explored. Thus, the overall aims of this study were to explore the effects of RFD on teamwork in IPSE for medical and nursing students:

1. Explore how healthcare students reflect on complexity and resilience in RFD through qualitative analysis of dialogue in debriefings.
2. Assess the effect of RFD in IPSE on team performance using the Team Emergency Assessment Measure (TEAM).
3. Obtain a deeper insight into the effects of RFD in IPSE on teamwork using mixed methods.

## Methods

### Design

**Fig. 1:**
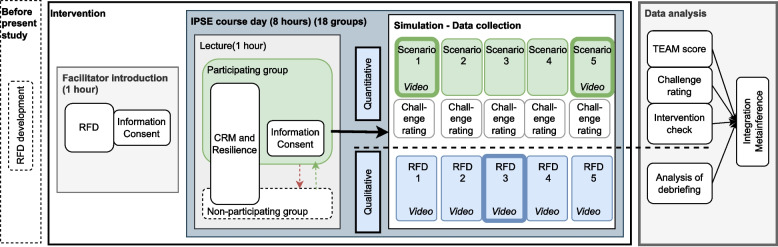
Intervention, data collection, and analysis for the study exploring the effect of resilience-focused debriefing (RFD) in interprofessional simulation-based education (IPSE) using mixed methods. The highlighted scenarios 1 and 5 form the basis for the quantitative analysis. The highlighted RFD 3 forms the basis for the qualitative analysis

A convergent mixed-method intervention design was used for this study [20]. We applied a pragmatic worldview [21] and drew on theories of complexity and resilience described above. To get a comprehensive understanding of the impact of RFD in IPSE we used a mixed methods study to explore both how the use of RFD affected the topics discussed in debriefings and its effects on team performance [22]. The quantitative and the qualitative data were analysed separately before the data were compared, contrasted, and synthesized to reveal collective insights. The study design is depicted in Fig. 1.

### Setting and participants

The research participants included nursing and medical students from the University of Gothenburg, Sweden, who were in their final years, and attended a mandatory IPSE course at Simulation Centre West, Sahlgrenska University Hospital, Gothenburg, Sweden. Prior to this, nursing and medical students completed one full day of interprofessional education, which comprised of lectures and discussion groups at the start of their programs. The research participants also included facilitators on this course. On one day, 2–4 groups of 6–10 students took part in five scenarios with subsequent debriefings (3–5 students being active while the rest observing). Debriefings were held by two facilitators, one nurse and one physician, to mirror the interprofessional composition of the student group. The two facilitators took turns serving as the primary facilitator during the day (See Additional file 1 for detailed information).

A total of 27 facilitators who were set to facilitate during the period of data collection were invited to participate in the study. All had previously participated in facilitator courses lasting at least 3 days. The course administration assigned facilitators and students to groups. Seven facilitators with limited experience (< 5 days) were excluded. One facilitator declined for personal reasons, and four were unable to participate due to logistical reasons. Thus, 15 facilitators took part in the study (Table 1).

**Table 1:** Facilitator demographics

	Facilitators (*n* = 15)
Age, mean (SD), median (min–max)	45.4 (8.9), 46 (28–59)
Women, n (%)	12 (80)
Men, n (%)	3 (20)
Nurses, n (%)	8 (53)
Physicians, n (%)	7 (47)
Previously facilitated in SBE, days, mean (SD), median (min–max)	53.1 (68.0), 20 (5–250)
Number of debriefings per facilitator in present study, mean (SD), median (min–max)	13.7 (8.8), 15 (5–30)

*SBE* Simulation-based education, *SD* Standard deviation

Students assigned to groups where both facilitators were already included as research participants, were invited to participate in the study. Of a total of 149 students who were initially asked to participate, seven nursing students and four medical students declined. The main reason for declining participation was a concern that the student’s performance could be impaired if recorded on video. If invited students declined to participate, they were replaced with volunteers of the same professions from a non-participating group. Thus, a total of 149 students in 18 groups participated, with average of 8.3 students in each group (Table 2).

**Table 2:** Student demographics

	Students (*n* = 149)
Age, mean (SD), median (min–max)	27.7 (6.6), 26 (21–59)
Women, n (%)	116 (78)
Men, n (%)	33 (22)
Nursing students, n (%)	73 (49)
Medical students, n (%)	76 (51)
Previously participated in SBE, days, mean (SD), median (min–max)	2.3 (1.1), 2 (0–10)
Times students were active in scenarios in present study, mean (SD), median (min–max)	2.4, (0.6), 2 (1–4)

*SD* Standard deviation

### Intervention

The participating facilitators were taught how to perform RFD during a 1-h, one-on-one online introduction with the first author (TNA), typically during the week preceding their first IPSE Day. The introduction covered the characteristics of complex systems and resilience. A study-specific RFD script (Additional file 2) was used as an aid for the facilitators. The script was developed from preliminary findings from previous studies [16, 17]. The script was structured according to the three-step format of description-analysis-application [23, 24]. In addition, four areas were stressed explicitly: 1) asking about contributions, 2) asking about challenges, 3) asking about expressions of complexity, and 4) asking for concretization of lessons learned. By the end of the introduction, the participating facilitators typically expressed that they had grasped the major ideas of the RFD approach. During data-collection the facilitators had opportunity to pose clarifying questions to first author.

For the students, the course day started with an interactive lecture on crisis resource management (CRM) [14] and complexity theory, covering concepts such as emergence and resilience.

No changes were made to the learning goals, content and format of the existing mandatory course, nor the existing difficulty progression of teamwork challenges in the scenarios (See Additional file 1 for details). However, to avoid bias in the subsequent external teamwork rating, the order of scenarios 1 and 5 was randomized for each group before the course day. To maintain the progression of difficulty, alternative variations of scenarios 1 and 5 were developed, with the teamwork challenges swapped, to be used in groups, where scenario 5 was run before scenario 1.

The order of students’ participation during the day was pre-scheduled according to a random identification number assigned to each that, so that the same four students who participated in scenario 1 also participated in scenario 5.

### Data collection

After conducting an IPSE pilot course day, data were prospectively collected between February and November 2023.

Qualitative data:

1. Nine video recordings of debriefings (approximately 5 h, average length 34 min (range 28–40)) for the intervention check and qualitative analysis.

Quantitative data:

2. Thirty-six video recordings of scenarios 1 and 5 (approximately 11 h, average length 19 min (range 8–25)) for rating TEAM performance.

3. Students’ ratings of their perceived degree of challenges after each scenario.

#### Intervention check

Scenario 3 debriefing transcripts from the first nine groups were chosen to assess the facilitators’ use of RFD by counting occurrences of each of the 18 specified debriefing techniques/questions (marked by numbers 1–18 in the RFD script in Additional file 2).

#### Qualitative data

All video recordings of debriefings from the first nine groups were transcribed verbatim (45 debriefings, totalling approximately 24 h). The video format was chosen to identify the profession and role of the speaker, i.e., facilitator, active in the scenario, or observer. Of these 45 debriefings, data from scenario 3 served as the basis for the qualitative analysis. Scenario 3 was chosen because it was the scenario in which students encountered the highest degree of perceived challenges according to their ratings. Thus, scenario 3 would provide the best basis for analysing how RFD was used when challenges were encountered.

#### Quantitative data

##### Team emergency assessment measure

TEAM [25] is an internationally established instrument for rating team performance that has shown evidence of validity and reliability in different healthcare contexts [26, 27], including in medical emergencies, and has been translated into Swedish [28]. It includes 12 items (Table 3), of which 11 are rated on a 5-point Likert scale (0 = never/hardly never; 1 = seldom; 2 = about as often as not; 3 = often; 4 = always/nearly always), and the 12th item is rated on an 11 point scale intended as an overall “gut reaction” of the team’s performance [25]. Summary scores are calculated for the categories *Leadership*, *Teamwork,* and *Task management,* and for items 1–11 (total sum score range 0–44).

**Table 3:** TEAM categories, elements, and items

Categories (score)	Elements	Items
Leadership (0–8)	Leadership control	1. The team leader let the team know what was expected of them through direction and command
		2. The team leader maintained a global perspective
Teamwork (0–28)	Communication	3. The team communicated effectively
	Co-operation and co-ordination	4. The team worked together to complete the tasks in a timely manner
	Team climate	5. The team acted with composure and control
		6. The team morale was positive
	Adaptability	7. The team adapted to changing situations
	Situation awareness (perception)	8. The team monitored and reassessed the situation
	Situation awareness (projection)	9. The team anticipated potential actions
Task Management (0–8)	Prioritization	10. The team prioritized tasks
	Clinical standards	11. The team followed approved standards/guidelines
Overall (0–10)		12. Global rating of the team’s performance

Adopted from Cooper et al. [25]

Three external simulation research experts – two consultants in anaesthesiology and intensive care (CE, JC) and one intensive care nurse (KJ) – from two other Swedish universities rated the recorded videos of scenario 1 and scenario 5. The raters had a total of 52 years of experience in simulation-based education. Two raters (KJ, JC) had previous experience using TEAM, and the third rater (CE) had experience with other teamwork rating systems. Before rating the videos, the raters and the first author rated two scenarios from the pilot data independently and then discussed their ratings to calibrate the rating process. Measures to blind the raters were made as described above. To complete the blinding procedure, all names of files and timestamps were changed in the videos so raters could not identify at what time of the day the scenarios were run.

##### Rating of perceived challenges

Using a study-specific 11-point numerical rating scale, the individual students who were active in the scenario rated the degree to which they encountered challenges in each scenario (“To what extent did you experience challenges in the scenario?”, rated as 0 = not challenging at all; 10 = extremely challenging). This scale was handed out by facilitators immediately after each scenario before beginning the debriefing.

### Data analysis

Two data strands were subjected to qualitative analysis of debriefing transcriptions inspired by topic analysis, because it focuses on how topics emerge and evolve during interaction and how participants construe shared meanings [29] and quantitative analysis with a prospective non-randomized blinded pre-post design using TEAM [25] for rating team performance in video-recorded scenarios; and the students’ ratings of the extent of perceived challenges in the scenarios.

#### Intervention check

In the debriefing transcripts of scenario 3, the facilitators’ questions were identified and labelled by TNA according to the 18 debriefing questions/techniques (or variations thereof) found in the script (Additional file 2) using Microsoft Excel. This served as a basis for quantifying how the facilitators in each group used each of the RFD techniques.

#### Qualitative analysis

The analysis of debriefing transcripts was based on the four main areas of the RFD script and performed by TNA, LO, HR and PA:

1. Asking about contributions
2. Asking about challenges
3. Asking about expressions of complexity
4. Asking for the concretization of lessons learned

These four areas were used as criteria to identify discussions (the questions asked by facilitators and how the students responded to them). Based on the above criteria, topical episodes were identified, i.e., “sequences of talk which are internally topically coherent, but which can be identified (at a reasonable level of reliability) by their boundaries, which demarcate them from adjacent episodes” [29]. Each topical episode was discussed within the research group regarding how the dialogue related to RFD, on how RFD was instrumental in addressing complexity and resilience, and what the typical and atypical characteristics of these dialogues were. These findings were described, condensed, and presented in short narratives with citations. Citations were translated from Swedish to English by TNA.

#### Statistical analysis

Nominal data are presented as numbers (n) and percentages (%), and continuous data are presented as means (standard deviation, SD; 95% confidence interval, CI), medians, and range (minimum–maximum). Statistical analyses were performed using IBM® SPSS® Statistics, version 29 (IBM, Armonk, NY, USA). The first 11 items of TEAM are presented as a mean value of the three raters’ assessment of each scenario for each of the 18 groups. The students’ ratings of their perceived challenges (numeric rating scale) were analysed as a continuous variable. For comparisons between scenario 1 and scenario 5, a paired t-test was used for continuous variables. The main results for continuous variables were the mean difference between scenario 1 and scenario 5 with 95% CIs and p-values. All tests were 2-tailed, and a statistical significance level of 5% was set.

#### Integration

All findings, both quantitative and qualitative, were compared, contrasted, interpreted and discussed within the research group, and presented as integrated results [20].

## Results

### Analysis of reflections in debriefings

#### Intervention check

Sixteen of the 18 specified RFD questions/techniques in the RFD script were used at least once in the nine debriefings of scenario 3. Seven (median, range 3–11) different debriefing questions/techniques were used in each debriefing at least once. The facilitators used a debriefing question/technique 13 times (median, range 5–26) in each debriefing. The most frequently used debriefing question/techniques were “Address the team members: What is your perspective on this?” (*n* = 20); “Tell me in what way you contributed to the successful teamwork and treatment of the patient?” (*n* = 18); “Did you have any challenges?” (*n* = 15); and “How did you solve it” (*n* = 15). However, “How did you manage to get out of it?” and “Discuss the pros and cons of working strictly according to guidelines or with deviations” were not used at all.

#### Qualitative analysis

Descriptions and interpretations are presented for typical dialogues relating to the four main areas of the RFD script. These illustrate in what ways the debriefing questions/techniques in the script helped facilitators guide the students’ reflections to uncover the complexity of situations and instances of resilient performances in more detail.

#### Asking about contributions

When students were asked how they contributed to the teamwork and the positive outcome for the patient, they shared examples that led to explorations of how concrete actions came about and the consequences these actions had for the scenario’s outcome.

When a student initially had struggled to recall and reflect on his contributions, follow-up RFD questions were used to unpack concrete step-by-step actions that helped the team manage the situation successfully.

FACILITATOR 1: [Nursing student 1], in what way did you contribute to solving the case?

NURSING STUDENT 1: Nothing, I think. I felt really lost in there.

FACILITATOR 1: Did you really do nothing?

NURSING STUDENT 1: Did I do something? I measured the blood pressure, I talked to the patient for a very long time, I called the doctor.

(three turns omitted)

FACILITATOR 1: The rest of you. In what way did you perceive [nursing student 1] contributed?

MEDICAL STUDENT 1: You did something all the time.

MEDICAL STUDENT 2: And when I didn't tell you what to do, then you said, “Should I set a peripheral venous catheter?”. And that's great, because I was standing there and I was completely blank, and it's great that someone asks, “Should I do this?”. (Group 9)

This example shows how a self-critical student can be encouraged to look more closely at how his/her actions contributed to managing the case. This was achieved by prompting students to look for exceptions to the initial impression of failure, which in turn led to recollections. How these actions were helpful became evident when the facilitator asked for perspectives from the other team members. The other members of the team clearly testified to how the actions had helped them to manage themselves and the case. Questions about contributions served to deepen insights into what went well and how success was achieved. Although this and similar episodes could be seen as demonstrations of resilience, they were not explicitly conceptualized as such in the debriefings.

#### Asking about challenges

Answers about challenges led facilitators to explore what events led up to the perceived challenges and what the students did to overcome the challenges. The following example recounts a scenario with challenges attributed to a combination of personal impressions of “messiness”, uncertainty, and unexpected events.

FACILITATOR 1: Did you have any challenges?

NURSING STUDENT 1: It feels like I forgot everything. I couldn't focus as I wanted; it was very confusing to me.

FACILITATOR 1: How did the rest of you perceive it? NURSING STUDENT 2: The same.

MEDICAL STUDENT 1: Yes, I thought it advanced really well until we found the bleeding, and then it got messy. And there was a lot in my head. I felt, well, yes, ok, that’s good, then we can put pressure on. But then, dammit, we couldn’t find the compresses. And then when I called for help, I got an unpleasant senior doctor on call in the phone. So, then it got a bit messy there. I think it was after that we sort of tied it together a bit and then came up with what it was.

FACILITATOR 1: And how did you do that?

MEDICAL STUDENT 1: By saying that it was messy and that we summarized what we had found, and then we moved on.

NURSING STUDENT 1: We communicated well with each other anyway, I think. (Group 5)

This example illustrates how a nursing student and a medical student felt stuck when the team discovered seemingly significant bleeding. The inability to immediately find a way to stop the bleeding and not getting the advice sought produced frustration. The facilitator’s questions on challenges encouraged the students to acknowledge and describe the complexity and uncertainty of the situation. Further, the question on solutions made it possible for them to describe their successful management within the scenario, i.e., bouncing back from a setback. The facilitator did not go on to ask the students what they should have or wished they had done. Rather, the emphasis was on how the students managed the scenario successfully despite not being in total control. This exemplifies an instance of resilience capacity in response to a complex situation, although it was not labelled in these terms. Thus, debriefing could make the students more aware of their own ability to manage challenging situations and tolerate “messiness” and surprises.

#### Asking about expressions of complexity

The RFD script provided suggestions to look closer at specific expressions of complexity and resilience, such as “messiness”, uncertainty, variations in demonstrated solutions, not adhering to guidelines, and hindsight bias. While there were few discussions on adherence to guidelines, discussions on nuances on how to carry out CRM principles were allowed. For instance, team members argued that coordinating their tasks by thinking aloud replaced the need for explicit leadership as recommended in CRM.

In the following example, the facilitator had the students explore the meaning of “messiness” and the consequences of expressing this within the scenario.

FACILITATOR 1: I’m thinking specifically of you saying out loud and clear, I’m feeling a bit messy. Can we go through it? What did that lead to?

MEDICAL STUDENT 1: But then we made some progress because that’s when we started putting pressure on the wound, I don’t remember the exact time, actually.

NURSING STUDENT 1: I also felt it was messy, and I didn’t know which letter we were on. I said, “Let’s start from the beginning”, and then we started from the beginning.

MEDICAL STUDENT 1: …it’s not that I feel that we as a team failed completely. It was more that I don’t like to feel the feeling of uncertainty.

FACILITATOR 1: Because you did solve the case. Why do you think that is?

OBSERVING NURSING STUDENT: …you couldn’t find compresses and stuff, and then it was like nobody knows where they are, and then you can’t ask each other, and in this uncertain situation…it’s a bit like because nobody knows, nobody moves forward either. … But in the end, you didn’t see if it was a towel or a blanket or whatever, so you just fixed it (with the towel). (Group 5)

This form of questioning shows how the facilitator used the term “messiness” as a cue to explore a complex situation. The facilitator further asked about the causes and the outcome of the situation, which revealed that several students found it “messy”. In addition, the facilitator’s questions drew attention to the complexity of the situation itself, and how an emergent uncertainty about how to treat a newly discovered bleeding wound and not finding compresses could contribute to their experiences of “messiness”. Further, the question about “messiness” brought attention to how the students adapted by taking a pause and becoming aware of the situation by using a CRM key point, “Re-evaluate often”. After this, the students re-evaluated, moved on together, and finally, they were able to resolve the situation by applying an alternative solution. Whilst this series of actions by the team can be seen as clear examples of resilience capacities, they were not explicitly addressed in these terms.

#### Asking for concretization of lessons learned

Concretization was mainly used when encouraging students to think about *concrete* personal action plans in the *next scenario* in the application phase of the debriefing. Such concretizations could lead to concrete answers – for example, when one student commented, “When I get confused, I shall stop and take a little pause to breathe”. However, the use of follow-up questions for the concretization of vague action plans was limited.

Alternatively, instances of facilitators rephrasing and summarizing dialogues on complexity and resilience were observed.

FACILITATOR 1: So, I get that you thought that the behaviour was very systematic and calm despite perhaps stress anyway?

NURSING STUDENT: Exactly, it wasn’t like this, just stop and panic, but something was done all the time, now we know, now we’ll call, it was like that. (Group 7)

This way of briefly summing up provides an example of how a facilitator can address resilience by directing students’ attention to how they were able to handle and adapt to complex situations despite experiencing hardship.

### Assessment of team performance

Based on the TEAM rating data, there were no significant differences between scenario 1 and scenario 5 for the individual category scores or the total score (Table 4).

**Table 4:** Team emergency assessment measurement scores based on the mean of the three raters’ scores for categories, for overall, and total score for scenario 1 and scenario 5, and the difference between them

	**Scenario 1 (*n* = 18)**	**Scenario 5 (*n* = 18)**	**Δ Scenario 5–1 (*n* = 18)**	
	Mean (SD)	Mean (SD)	Mean (SD)	***P***-value
	Median (Min; Max)	Median (Min; Max)	[95% CI]	
Leadership	5.5 (0.8)	5.2 (1.3)	−0.3 (1.3)	*p* = 0.40
	5.3 (4.0;6.7)	5.5 (2.7;7.0)	[−0.9;0.4]	
Teamwork	20.0 (2.3)	19.5 (2.3)	−0.5 (2.9)	*p* = 0.46
	20.3 (16.3;24.0)	19.8 (14.7;23.0)	[−2.0;0.9]	
Task Management	5.5 (0.7)	5.5 (0.7)	0.0 (1.1)	*p* = 0.94
	5.7 (3.7;6.3)	5.7 (3.7;6.3)	[−0.5;0.5]	
Overall	6.9 (0.8)	6.6 (0.9)	−0.3 (1.0)	*p* = 0.22
	7.0 (5.0;8.0)	6.8 (4.7;8.0)	[−0.8;0.2]	
Total	31.0 (3.4)	30.3 (3.7)	−0.8 (4.6)	*p* = 0.49
	31.5 (25.0;37.0)	31.5 (23.0;35.7)	[−3.0;1.5]	

*SD* Standard deviation, *CI* Confidence interval

**Fig. 2:**
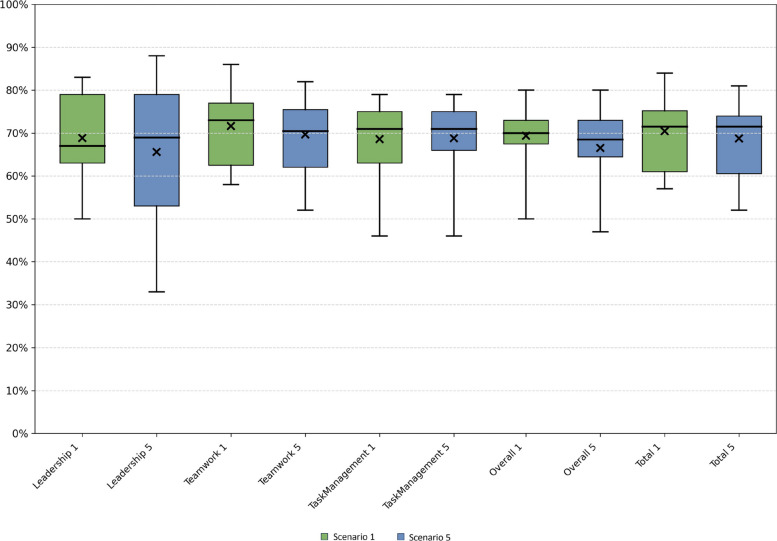
Box plots showing distributions of the Team Emergency Assessment Measurement scores as percentages of the maximum score in scenario 1 and scenario 5 for the category, overall, and total scores. The boxes span the first and third quartiles, with a line indicating the median, and X the mean. Whiskers show minimum and maximum values

The TEAM scores for categories, overall score, and total score were all approximately 70% of the maximal score in both scenario 1 and scenario 5 (range 66.7–72.6%) (Fig. 2).

### Rating of perceived challenges

The students’ (*n* = 149) perceived challenges decreased from scenario 1 to scenario 5 by 0.8 points (mean, range −7 to 4, *p* = 0.003) (Table 5).

**Table 5:** Students’ ratings of the degree of perceived challenges they encountered in the scenarios

	Scenario 1	Scenario 2	Scenario 3	Scenario 4	Scenario 5	Δ scenario 5–1	
	Mean (SD)	Mean (SD)	Mean (SD)	Mean (SD)	Mean (SD)	Mean (SD)	***P***-value
	Median	Median	Median	Median	Median	[95% CI]	
	(Min; Max)	(Min; Max)	(Min; Max)	(Min; Max)	(Min; Max)		
Students^*^, n	72	73	66	56	72	70	
To what extent did you experience challenges in the scenario?	5.7 (1.5)	5.3 (1.6)	6.1 (2.1)	4.5 (1.8)	5.0 (1.8)	−0.8 (2.1)	*p* = 0.003
	6	5	6	4	5	[−1.3;0.3]	
	(2;9)	(1;10)	(1;10)	(0;8)	(0;9)		

*SD* Standard deviation, *CI* 95% confidence interval

^*^Only the students who actively participated in each scenario rated the scenario. Two students did not participate in scenario 1 and scenario 5

### Integration of the findings from different data sources

The qualitative data showed that RFD questions/techniques aided facilitators to guide the students’ attention to complexity and how they managed it by applying CRM principles and performing resilient actions (e.g., attunement, adaptive leadership) both as individuals and as teams. Applying RFD brought attention to how the students were able to succeed despite the difficulties they encountered. Further, despite achieving acceptable performance according to TEAM’s ratings, analysis of debriefings revealed that the students did not initially recognize their own successful contributions to interprofessional teamwork. The decrease in the degree of challenges experienced may indicate that students developed a greater tolerance for complexity, such as “messiness” and uncertainty, through their reflections in the debriefings. Despite these findings, the quantitative data showed that there was no difference in team performance as assessed by TEAM during the IPSE course day. Additionally, the intervention check of the qualitative data demonstrated that there were few examples of concretizations of lessons learned, leaving out formulations of feasible action plans, particularly in the application phase (Table 6).

**Table 6:** Interpretation of qualitative and quantitative findings

Qualitative findings	Quantitative findings
• RFD guides the students’ attention to: o the complexity of situations and its effects on teamwork o that complexity was managed successfully despite individual and team-related difficulties o how complexity was managed successfully by responding to emerging needs in the situation o the effects of their actions on teamwork and patient outcome • Few examples of concretizations of lessons learned	• Number of RFD debriefing techniques used varied o Few instances of concretizations • Team performance was acceptable • No change in team performance • Students experienced challenges to a significant lesser extent in the last scenario

## Discussion

In this convergent mixed-method intervention study, we found that RFD helped facilitators guide the students’ attention to the complexity of teamwork and how to successfully manage acute dynamic situations by adapting CRM principles and performing resilient actions (e.g., attunement, adaptive leadership), both as individuals and as teams.

Analysis of the debriefing dialogues indicates that RFD questions and techniques help students to discuss their ability to perform successfully despite expressions of negative self-perceptions. Thus, debriefing helps students calibrate their self-perception by learning from their own lived experiences of achieving success [3, 4]. We found that facilitators and peers perceived a student’s contribution to interprofessional teamwork as more substantial than the student did. This is reflected in the acceptable level of the total TEAM score comparable of Baker and Maignan [30, 31]. Although we did not investigate the dynamics behind these findings, they might stem from differences in perception, assessment, or the reluctance to discuss one’s own performance in a positive light [32]. While the focus on strengths is likely a positive experience for the student as a person, it could also have a positive effect on the team and the patient. Thus, RFD may help foster a supportive team climate. Furthermore, this might indicate that the team perspective is more important than the individual for the outcome of the patient.

Our study indicates that CRM principles can be adapted to the situation at hand by emphasizing how to be resilient and specifying solutions to overcome challenges. This notion aligns with a central aim of CRM, to handle complexity and dynamic situations, e.g., by adapting or reevaluating [33–37]. In addition, RFD can bring attention to concrete actions that highlight nuances of CRM, e.g., attunement, adaptive leadership, tolerating “messiness” and uncertainty, and deviation from guidelines when necessary. Such “resilient skills” or performances have been presented in a number of studies focusing on how to handle complexity and resilience on individual, team, and organizational levels [36, 38–45]. On this background, we hypothesize that RFD could work as a pedagogical tool to link the practical use of CRM to the theoretical foundations that form the basis for resilience. Drawing attention to the complexity of a situation in debriefing is not a novel idea. Debriefing methods such as debriefing with good judgment, PEARLS, and TeamGAINS [46–48] recommend doing so, albeit in slightly different ways. However, we argue that the use, training, and evaluation of these debriefing methods is often done with a Safety-I mindset, i.e., to focus on identifying mistakes and correcting them to avoid them in the future. Taking a resilient or Safety-II mindset, as implied in RFD, lets us expand students’ understanding of CRM principles by exploring how specific actions promote resilience, for example, how effective leadership also comprises adaptation. We contend that such an approach could prepare students for the complexities of clinical work in a more meaningful and realistic way.

The analysis of the debriefings shows how RFD can offer an exploratory approach when the opening questions on contributions and challenges are succeeded by follow-up questions. This is, to a large extent, centred on curiosity – rather than blame and accusations – and oriented by a learning mindset that goes beyond issuing corrections [4, 48, 49]. This also emphasizes that RFD does not simply consist of a number of debriefing questions or techniques. The facilitators must be willing to put extra time into uncovering complexities, perspectives, multiple contributing factors to outcomes, and recovering from setbacks.

Although RFD seemed to impact the students’ reflections on teamwork in the scenarios, there was no effect on observed interprofessional teamwork in the quantitative analysis, as measured by the pre-post difference in team performance with TEAM. There may have been several reasons to account for this discrepancy.

### Progression of difficulty

Given that scenario 5 was intentionally designed to be more challenging than other scenarios, the unchanged TEAM scores – combined with students’ significantly lower perception of challenges – may suggest an improvement in team performance. However, this interpretation cannot be confirmed with certainty based on the current study design, which aimed to explore an intervention in an existing practice, with only a few changes permitted. To measure improvement, inclusion of comparable scenarios would be preferable.

### Previous student education

The TEAM score was relatively high in the first scenario [30, 31, 50]. This may be an indication that the lecture covering CRM principles and resilience, as well as previous days of simulation with some degree of CRM, prepared the students to apply this knowledge quite readily in the first scenario.

### Education of facilitators

The intervention check showed that the facilitators used RFD to a varied degree. We saw few examples of concretization, particularly in the application phase. In a previous study [17], facilitators found that concretization was crucial, as attention placed on complexity and resilience by itself may not be sufficient to provide students with an action plan forward [51]. The consequence of omitting concretization may have resulted in poor translation of debriefing insights into observable improvements in team performance.

The one-hour one-on-one online introduction to RFD the week prior to the first IPSE-day setup was chosen because only a few facilitators were able to attend a 4-h workshop on RFD, which was originally planned. Consequently, our findings highlight the need for further research involving more systematic strategies for educating facilitators in debriefing that address complexity and resilience.

### Assessing resilience

Although the TEAM instrument includes items and behavioural markers suggestive of resilience—such as adaptation—it is largely grounded in a Safety-I paradigm, emphasizing normative compliance and deviations from predefined guidelines. Similar limitations apply to other widely used tools, including Anaesthetists’ Non-technical Skills (ANTS) [52], Team Performance Observation Tool (TPOT) [53], and the Ottawa CRM global rating scale [54]. These instruments may fail to capture improvements regarding complexity and resilience; For instance, constructs such as “tolerance of uncertainty” are difficult to observe directly. Moreover, such rating tools are traditionally designed to assess behaviours more or less in isolation. Longer processes or outcome is not considered [55]. This means that observed difficulties that were later resolved without any negative outcomes for the patient still count negatively. Several authors have described the need for such instruments to incorporate behavioural markers for characteristics such as team resilience and adaptive coordination [36, 56–59]. We argue that these considerations underscore the importance of further research on evaluating teamwork in the light of complexity and resilience.

In addition, the students reported experiencing fewer perceived challenges during the final scenario. This may suggest a developing tolerance for expressions of complexity, such as uncertainty and “messiness”. However, we cannot exclude the possibility that students learned “to play the game” of simulation better, gaining a clearer understanding of expected behaviours and responses. They might have also become familiar with the simulation environment, each other, and the facilitators. Finally, the potential influence of cognitive or emotional fatigue over the course of the day may also be considered.

### Strengths and limitations

This study meets calls for theory-driven research in SBE [60] and research on practical applications of resilient healthcare theories [8, 61]. We took guidance from the six core quality criteria of mixed methods research by Hirose et al. in order to ensure the methodological quality [22]. To ensure trustworthiness and dependability in the qualitative research process, strategies were employed in accordance with the criteria established by Lincoln and Guba [62]. The data analysis was first performed independently by each of the authors, TNA, HR, LO, and PA, and then in collaboration to identify and conduct a closer analysis of representative examples. The authors comprised researchers within nursing, medicine, and the learning sciences with various professional backgrounds. The participating facilitators were diverse in gender, professions, and specialties. The qualitative data analysis was performed on a subset of debriefings (scenario 3), which was considered to yield sufficient information power to answer the research questions [63, 64]. We collected data prospectively during two semesters in 2023 with 149 students in 18 groups. No power calculation was performed in this mixed-method study, but the sample size is comparable with other studies using TEAM for assessing team performance [31, 50].

## Conclusions

Techniques in RFD can be used to increase healthcare students’ attention to the complexity of interprofessional teamwork in acute dynamic situations. Furthermore, applying RFD can help students recognize and learn from successful actions, both as individuals and as teams. Bringing to attention how unpredictable situations are actually managed, step by step, by performing resilient actions, may potentially improve interprofessional teamwork. In addition, this approach could possibly be a gateway to teach medical and nursing students about the complex nature of healthcare in general. Furthermore, these findings exemplify how the practical application of complexity theory and resilience can improve healthcare team education to enhance patient safety and the quality of care. While RFD showed promising results in the current study, further research is needed on facilitator education in the use of RFD to fully assess its impact on teamwork in simulation-based education. Additional research is warranted on the development of instruments for measuring team performance that are sensitive to various aspects of resilience, such as registering recovery from setbacks and taking outcomes into account.

## Supplementary Information

Additional file 1

Additional file 2

## Data Availability

The datasets used and analysed during the current study are available from the corresponding author on reasonable request.

## Abbreviations

IPSE: Interprofessional simulation-based education
RFD: Resilience-focused debriefing
TEAM: Team emergency assessment measure
CRM: Crisis resource management
