# Supplementary material for: Exploring effects of resilience-focused debriefing on reflection and teamwork in interprofessional simulation-based education – a mixed method study
Source: Adv Simul (Lond). 2025 Dec 19;11:4. doi: 10.1186/s41077-025-00398-4 (PMC12831296; doi:10.1186/s41077-025-00398-4)
Supplement: Supplementary file 1 — Additional file 1 [file 41077_2025_398_MOESM1_ESM.pdf]

Additional file 1

Detailed description of the setting

During their final semester, around 200 nursing students and medical students at the University of Gothenburg, Gothenburg, Sweden, participated in a mandatory one-day IPSE course. The learning objectives were to train the algorithm of ABCDE (airway, breathing, circulation, disability, exposure/environment), principles of crisis resource management (CRM), and interprofessional collaboration. The course comprised an interactive lecture on CRM, an introduction to the simulation environment, and 5 scenarios, each lasting about 25 minutes. Each scenario was followed by a debriefing lasting about 30 minutes. Groups of approximately 8 students, 4 of each profession, were formed. Each student participated in 2-3 scenarios. The scenarios contained common patient conditions found in general hospital wards and primary care (exacerbation of chronic obstructive pulmonary disease, stupor caused by hypoglycemia, hypotension due to postoperative bleeding, postoperative sepsis, and ketoacidosis with comorbidities like hypertension or atrial fibrillation). The scenarios varied in teamwork challenges, timing of onset of patient deterioration, and presence of relatives, among others.

1. Scenario 1: The whole team of two nursing students and two medical students was sent to attend to a patient who was acutely ill at the emergency ward.
2. Scenario 3: Two nursing students were sent to a geriatric ward room to attend a disoriented elderly patient operated for a hip fracture the day before. They find low saturation and blood pressure. They get immediate help by calling two medical students.
3. Scenario 5: Two nursing students were sent to attend to a patient in a general practice ward and found a quite ill patient, who deteriorated. They needed to make two calls to get the two medical students to come, as they were engaged in other important matters.

Thus, the difficulties of the scenarios were designed to progress over the course of the day. While 3-4 students were active in the scenarios, the rest of the students in the group watched a live stream from an adjacent room. All students participated in the debriefing, which was conducted by two facilitators, a nurse and a physician, following the three-phase model by Steinwachs (description-analysis-application) .
