# Supplementary material for: Exploring effects of resilience-focused debriefing on reflection and teamwork in interprofessional simulation-based education – a mixed method study
Source: Adv Simul (Lond). 2025 Dec 19;11:4. doi: 10.1186/s41077-025-00398-4 (PMC12831296; doi:10.1186/s41077-025-00398-4)
Supplement: Supplementary file 2 — Additional file 2 [file 41077_2025_398_MOESM2_ESM.pdf]

Additional file 2

Resilience-focused debriefing

This is an example of how debriefing techniques that address complexity and resilience in simulation-based team training can be used sequentially as part of a script. This is not a guideline that should be followed strictly, but rather a suggestion for the use of the techniques (Amoroe et al., 2025). It is mostly thought of as an add-on to whatever debriefing model you usually use.

Background

According to criticism from Resilient Healthcare (RHC), one contributing factor to inadequate patient safety is that healthcare professionals are not sufficiently aware of the complexity and high degree of unpredictability in healthcare. Healthcare staff must constantly manage surprises, interruptions, and disturbances, which they often handle well—this ability is referred to as resilience. To gain a comprehensive understanding of this complexity, one must be aware of the phenomenon's existence and, during analysis, not only focus on what goes wrong but also on what goes right.

A number of therapeutic and organizational development techniques align with this perspective, arguing that effective development comes from learning from positive experiences and focusing on positive outcomes (e.g., solution-focused approach and appreciative inquiry).

Traditional interprofessional simulation-based education (IPSE) tends to focus on doing the right thing according to context-free general guidelines and algorithms, and on correcting deviations from these, without sufficiently considering variations and specific dynamic contexts. Therefore, there is a need to develop a method for IPSE—especially debriefing—that takes these factors into account.

These partially new debriefing principles are based on the traditional three-phase method by Steinwachs (description, analysis, application phase), which is widely used and taught by, among others, EuSim, CAMES, and the Simulation Center West, Gothenburg, Sweden. These principles adjust the traditional model to address the criticisms and suggestions mentioned above.

These questions or techniques have been developed through design-based research involving instructors and students in IPSE (Amoroe et al., 2023, 2025).

Execution

Lecture

Participants should be briefly introduced to the concepts of complexity, unpredictability, the problem of linear causality, and team resilience. Have the participants reflect on how these concepts relate to their own clinical experiences in small interprofessional groups. Make it clear that the upcoming scenarios possibly will place them in situations marked by uncertainty, ambiguity, dilemmas, and impressions of chaos. It is important to emphasize that the experience of uncertainty is a normal, expected part of clinical work. Such feelings often arise not from a lack of knowledge or skill, but from the inherent complexity or messiness of the situation itself. Remind the participants that Crisis Resource Management (CRM) principles are available to help bring clarity and structure to these challenging moments. Encourage participants to foster awareness, to adopt a constructive, solution-focused mindset rather than falling into self-critical devaluation.

Write overall learning objectives for the day at the top a whiteboard: For example: 1. ABCDE, 2. Communicate effectively, 3. Good teamwork, 4. Good leadership.

Briefing

- Give observers individual items to look for according to the learning objectives.

During scenario

- Note situations related to the learning objective, and be sure to note successes.
- Note situations where students seem to be uncertain, are about to make the wrong decision, make the wrong decision or correct already decided and/or executed decisions. Note how the team handles this.

Debriefing

Introduction

- Inform about the time available and the debriefing phases

Description phase

- Asking for the participants' feelings is optional, but we recommend it. Maybe give participants 10 seconds to think before they answer.
- Recognize/address challenges, ("so it was challenging...", "... We can come back to that") (=pearls/balloons).
- Maybe ask if the patient seemed realistic and address possible technical issues.
- Specific medical questions may be addressed separately here, but it is preferable to analyze the management of these uncertainties in the analysis phase.

Analysis phase

<u>Ask about contributions</u>

- ⇒ Addressing the whole team: Tell me in what way you contributed[1] to successful[2] teamwork and treatment of the patient.
  - ⇒ Addressing individuals when the topic warrants an individual perspective or the strengthening of a learning point for that individual.
    - ⇒ Why did it turn out this way?[3] Why did you do this? What strategies did you use?
      - ⇒ What did that lead to?[4] What effect did it have on the team? What significance did it have for the patient?[5]
        - ⇒ If addressing an individual, address the team members: What is your perspective on this?[6]
          - ⇒ Back to the same individual the team is talking about: What do you think when you hear this?[7]
- ⇒ Ask observers for their observations of what worked well.

<u>Ask about challenges</u> (It is possible to spend more time on challenges by bringing up challenges earlier, especially if it seems very important to the participants).

- ⇒ Addressing the whole team: Did you face any challenges?[8]
  - ⇒ Addressing individuals when the topic warrants an individual perspective or the strengthening of a learning point for that individual.
    - ⇒ Use pearls/balloons if present: You said earlier that x was a challenge, can you tell us about it?
      - ⇒ Why did it turn out in this way?[3]
        - ⇒ Did you get out of it? How did you manage to get out of it?[10] How did you solve it?[9]
          - ⇒ What did you want to achieve?[11] How would you like it to be?[12]
            - ⇒ Address team members: What are your views on what X says?[6]
              - ⇒ Address individual: What do you think when you hear this?[7]
            - ⇒ If you were to perform this scenario again, what would you do then?[12]
            - ⇒ If vague: How would that sound? How do you say that? What do you do then? When do you do it?[13]
- ⇒ Ask observers for their observations on what looked challenging or could have been done differently.

<u>On asking about complexity</u>, when it appears as challenges (or as successful contributions)

- Pay attention to expressions of messiness, uncertainty, and disturbing circumstances[14].
- Examine how these circumstances were handled: "What happened?", "How did you solve it?", "How did you solve it together?", and "What did it lead to (for the patient)?"
- Pay attention to separate PERSON and SITUATION: If a person was in a demanding situation: Validate: "It certainly was a difficult situation" and follow up with "What would you have needed in order to resolve this situation more satisfactorily?", "What did the rest of you do?", "Is there anything you or the others could do to make it easier?".
- Possibly discuss the pros and cons of working strictly according to guidelines or with deviations[15], e.g., working in parallel on several letters (ABCDE).
- Pay attention and contradict if someone blames themselves or draws conclusions from HINDSIGHT[16].
- Inform students that reality is messy and that the feeling of messiness may exist because the situation IS messy and dynamic.
- Possibly discuss the difference between learned knowledge, plans, the ideal world vs the unpredictable, complex real world. What is the significance of these different aspects?

Application phase

- Think for 10 seconds[17] about what concrete actions or strategies you should focus on or do more of in the next scenario[18]. (After the last scenario, say "in reality").
- If unspecific, undoable answers are provided, ask additional questions to get concrete answers ("... and how do you do it?", "how does it sound"?)[14]

If time allows, answers may be written on a whiteboard by the facilitator or on paper by the participants.

Amoroe, T. N., Rystedt, H., Oxelmark, L., Dieckmann, P., & Andrell, P. (2023). How theories of complexity and resilience affect interprofessional simulation-based education: a qualitative analysis of facilitators' perspectives. *BMC Medical Education, 23*(1), 717. doi:10.1186/s12909-023-04690-7

Amoroe, T. N., Rystedt, H., Oxelmark, L., Dieckmann, P., & Andrell, P. (2025). Resilience-focused debriefing: addressing complexity in interprofessional simulation-based education-a design-based research study. *Advances in Simulation, 10*(1), 25. doi:10.1186/s41077-025-00352-4

[1-18] Indicates specific RFD questions/techniques that was employed in assessing facilitators' use of RFD.
